# The quantification of southern corn leaf blight disease using deep UV fluorescence spectroscopy and autoencoder anomaly detection techniques

**DOI:** 10.1371/journal.pone.0301779

**Published:** 2024-05-15

**Authors:** Hashem Banah, Peter J. Balint-Kurti, Gabriella Houdinet, Christine V. Hawkes, Michael Kudenov

**Affiliations:** 1 Department of Electrical and Computer Engineering, North Carolina State University, Raleigh, NC, United States of America; 2 USDA-ARS, Plant Science Research Unit and Entomology and Plant Pathology Department, North Carolina State University, Raleigh, NC, United States of America; 3 Department of Plant and Microbial Biology, North Carolina State University, Raleigh, NC, United States of America; 4 NC Plant Science Initiative, North Carolina State University, Raleigh, NC, United States of America; Sakarya Uygulamali Bilimler Universitesi, TURKEY

## Abstract

Southern leaf blight (SLB) is a foliar disease caused by the fungus *Cochliobolus heterostrophus* infecting maize plants in humid, warm weather conditions. SLB causes production losses to corn producers in different regions of the world such as Latin America, Europe, India, and Africa. In this paper, we demonstrate a non-destructive method to quantify the signs of fungal infection in SLB-infected corn plants using a deep UV (DUV) fluorescence spectrometer, with a 248.6 nm excitation wavelength, to acquire the emission spectra of healthy and SLB-infected corn leaves. Fluorescence emission spectra of healthy and diseased leaves were used to train an Autoencoder (AE) anomaly detection algorithm—an unsupervised machine learning model—to quantify the phenotype associated with SLB-infected leaves. For all samples, the signature of corn leaves consisted of two prominent peaks around 450 nm and 325 nm. However, SLB-infected leaves showed a higher response at 325 nm compared to healthy leaves, which was correlated to the presence of *C. heterostrophus* based on disease severity ratings from Visual Scores (VS). Specifically, we observed a linear inverse relationship between the AE error and the VS (*R*^2^ = 0.94 and *RMSE* = 0.935). With improved hardware, this method may enable improved quantification of SLB infection versus visual scoring based on e.g., fungal spore concentration per unit area and spatial localization.

## Introduction

Southern leaf blight (SLB) is a corn disease caused by the fungus *Cochliobolus heterostrophus* (anamorph: *Bipolaris maydis*) [1]. Warm and humid weather conditions are suitable for *C. heterostrophus* to infect maize plants. The disease is a particular problem in warm humid regions such as Latin America, Africa, India, and the southern part of Europe [2]. This pathogenic fungus has two races: race T, and non-race T, known as race O. In 1970, the US Corn Belt suffered from an epidemic that caused 15% losses in corn yield due to the widespread plantation of corn plants sensitive to race T [3]. After the epidemic, race-T-resistant maize plants were planted, which reduced the spread of SLB in the US. Meanwhile, race O is still prominent in the US and China [4], and still causes production losses worldwide [5]. Therefore, the accurate quantification of the symptoms associated with SLB infection could mitigate the propagation of the disease and preserve the yield of corn production.

The conventional monitoring of healthy and diseased plants is conducted by visual examination of the symptoms [6, 7]. For fungal diseases, scientists usually rate plants based on the severity of infection [8]. These methods are time-consuming and somewhat dependent on the observer’s interpretation skills. In some cases, more sophisticated microbiological or molecular techniques have been utilized to quantify fungal infections, such as culture and colony counting [9] and polymerase chain reaction (PCR) [10]. Although these detection techniques are accurate, a faster, less laborious, and scalable method is needed to identify fungal infections in plants.

The use of optical detection techniques such as Fluorescence Spectroscopy (FS) has attracted interest in plant and food science due to its potential to identify small molecular structures and detect diseases even at early stages [11, 12]. Nevertheless, there are only a few studies that have utilized FS in determining fungal infection in plants [13]. FS and synchronous fluorescence spectroscopy (SFS), using 410 nm and 200-800 nm excitation wavelengths respectively, were used to detect stripe rust infection caused by *puccinia striiformis f. sp. tritici* in asymptomatic and symptomatic wheat leaves. In most cases, the blue-green fluorescence (BGF) increased while the chlorophyll fluorescence (ChlF) decreased with high infection rates [12]. Another study utilized laser-induced fluorescence (LIF) with 337 nm excitation wavelength to detect powdery mildew infection caused by *blumeria graminis f. sp. tritici* in presymptomatic wheat leaves [14]. By utilizing different fluorescence ratios for detection, the inoculated samples showed a continuous decrease in the blue-to-green ratio (F451/F522) and a significant increase in the green-to-red (F522/F687) and green-to-far-red (F522/F736) ratios 4 days after inoculation. Rot, caused by fungi and other species, was detected in apples and potatoes using fluorescence spectroscopy with 405 nm and 527 nm excitation wavelengths. Generally, the emission spectra of rotten samples showed differences in intensity, and sometimes phase, from the emission spectra of healthy samples [15].

FS has also been used extensively to detect aflatoxin, a toxin generated by *aspergillus flavus*, in different agricultural commodities. For instance, aflatoxin affected the intensity and phase of intrinsic maize grains spectra using one and two-photon laser-induced FS using 365 nm and 730 nm excitation wavelengths respectively [16]. In pistachio, laser-induced FS (LIFS) with 308 nm excitation wavelength was used to determine aflatoxin contamination through the rise of a small peak in the blue region coupled with a high peak in the green region of the spectrum [17]. Another study determined aflatoxigenic fungi contamination in peanut kernels using fluorescence spectroscopy and headspace-gas chromatography-ion mobility spectrometry (HS-GC-IMS). The contaminated samples showed an increase in fluorescence emission in the 415-440 nm and 500-510 nm ranges versus time [18]. Apart from analyzing the changes in the intrinsic emission spectra of plants, Fauzia et al. (2018) detected the fungal metabolite spectra of *aspergillus flavus*, *bipolaris oryzae*, and *fusarium semitectum* at 440 nm, 534 nm, and 510 nm respectively, on rice seed using fiber optic FS with 365 nm excitation wavelength [19].

Despite the successful use of FS for fungal detection in leaves, we exploited DUV lasers, lasers with output wavelengths between 224 nm to 280 nm, as an excitation source to quantify the phenotype associated with SLB infection in corn plants. According to Bhartia et al. (2008), using an excitation source below 250 nm is needed to clearly acquire the intrinsic fluorescence features of organic and biological materials [20]. To our knowledge, no previous efforts have utilized this technology for foliar phenotyping. A possible reason is the difficulties associated with the production of compact DUV lasers and light sources. The lifespan of laser systems that operate in the DUV range is low and their cost is high compared to other laser systems [21]. Nevertheless, Rasch et al. (2010) used DUV-FS with 280 nm excitation wavelength for detecting different mycotoxins in wheat grains, but the toxin emission spectra interfered with the intrinsic emission spectra of grains [22].

In this study, we propose the use of a non-destructive method to quantify the symptoms of fungal infection in SLB-infected corn plants using DUV fluorescence spectroscopy and autoencoder anomaly detection techniques. Here, we used a 248.6 nm excitation wavelength to acquire the emission spectra of healthy and SLB-infected leaves. Spectra of healthy and diseased leaves were then used to train an Autoencoder (AE) anomaly detection algorithm. This technique allows the automated quantification of SLB infection, instead of the visual scores, based on the phenotype caused by *C. heterostrophus* on the leaves’ emission spectrum. This can be a stepping point to correlate the optical emission spectrum of SLB-infected leaves to the presence of C. heterostrophus based on e.g., fungal spore concentration and spatial localization (as determined by microscopy) within the lesion.

## Materials and methods

### Cochliobolus heterostrophus cultures

**Fig 1:**
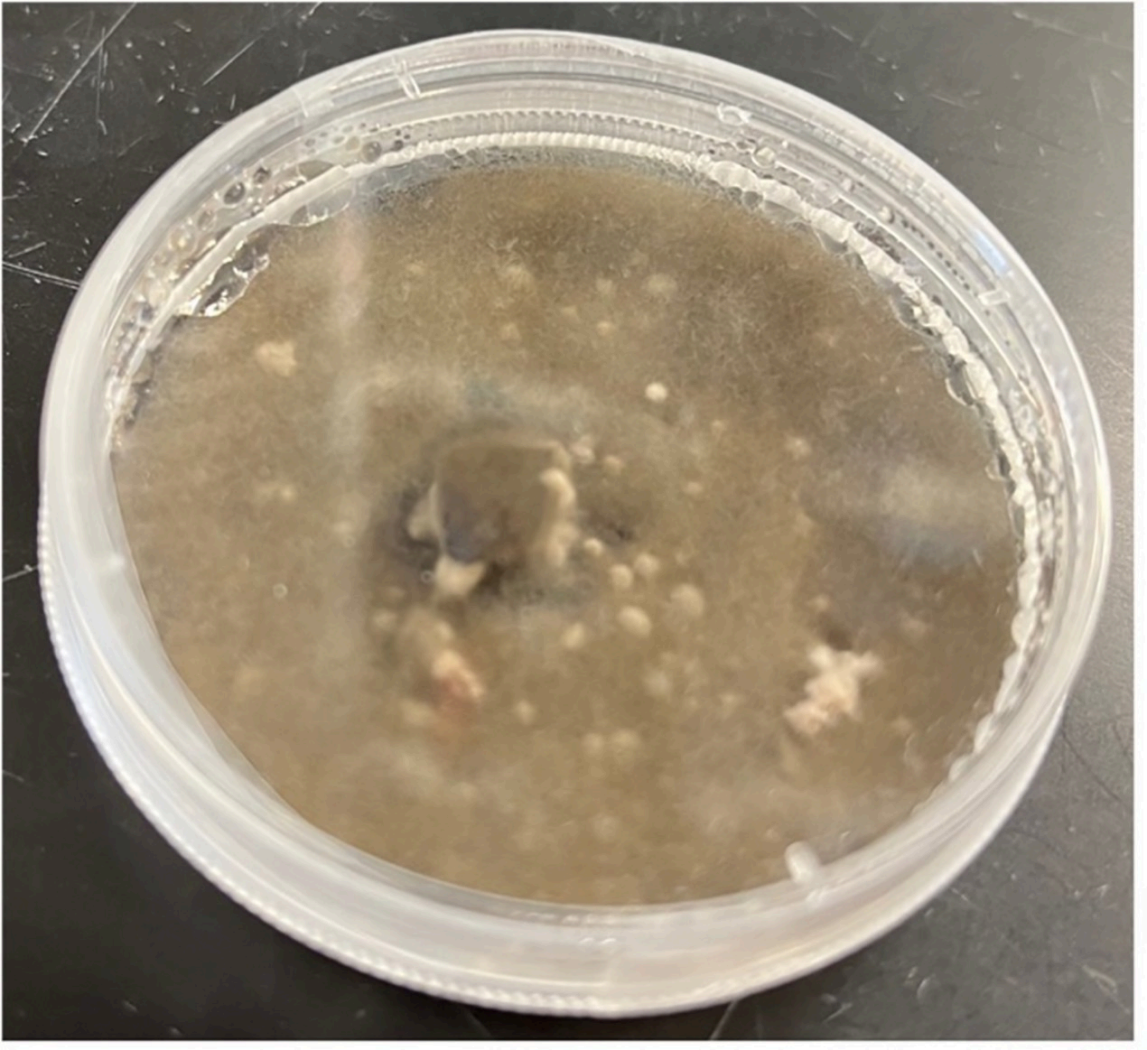
PDA plate cultivated with *C. heterostrophus*.

*Cochliobolus heterostrophus* was grown on the dehydrated format of potato dextrose agar (PDA) plates, which consisted of 1000mL of water, 4g of potatoes, 20g of dextrose, and agar powder. PDA plates served as a cultivation media for the fungus. Plates were inoculated with a fungal sample via an agar core from an active plate. Then, the plates were wrapped with parafilm and put in an incubator to grow at 25°*C* for 4 days, then left on the bench top under the light. The PDA plate cultivated with *C. heterostrophus* is shown in Fig 1. The Cochliobolus heterostrophus isolate used was 2-16 bm which has been described previously [23]. This isolate is of the mat 1-2 mating type and is of race O pathogenicity. The isolate was originally isolated from a commercial maize field in Wilkes County, NC in 1985.

### Field experiment

The field trials were conducted in the summer months, between June and August, over the years 2021 and 2022 at the Central Crop Research Station in Clayton, NC. Experimental units (lines) consisted of 2 m single-row plots with a spacing of 0.97 m, arranged in randomized complete blocks. Border rows were planted on all sides of the field. In Table 1, the lines are a mix of genetically diverse populations. Whereas, In Table 2, the lines assessed derived from a previously described population of lines that were near-isogenic (genetically identical) to the commonly-used maize line B73, the best-studied maize line [24]. This population was selected based on their variation in resistance to SLB [25].

**Table 1:** 2021 field experiment: Row-Code of a leaf sample; Line-Genetic population; Date-Scoring date; VS-Visual score given.

2021 Field Visual Scores							
Row	Line	Date 1	VS	Date 2	VS	Date 3	VS
06076	CML69	07/07	6	07/12	8	07/15	8
06079	CML247	07/07	8	07/12	8	07/15	8
03110	M0085	07/09	7.5	07/12	-	07/16	7.5
06078	CML228	07/07	7	07/12	8	07/15	7.5
06085	Ki3	07/15	8	07/18	7.5	07/21	7
06086	Ki11	07/15	8	07/18	7.5	07/21	7
03113	M0258 X Mo17	07/20	8	07/24	7	07/27	-
03106	M0116 x B73	07/20	7.5	07/24	6	07/27	-
06087	Ky21	07/15	5.5	07/18	4.5	07/21	5

**Table 2:** 2022 Field Experiment: Row-Code of a leaf sample; Line-Genetic population; Date-Scoring date; VS-Visual score given.

2022 Field Visual Scores							
Row	Line	Date 1	VS	Date 2	VS	Date 3	VS
11020	CML228/B73 NIL-1139	07/07	8	07/10	7.5	07/13	8
11042	CML247/B73 NIL-1197	07/07	7.5	07/10	7.5	07/13	7.5
11018	CML322/B73 NIL-1211	07/20	7	07/24	7	07/27	7
11034	CML247/B73 NIL-1128	07/07	7.5	07/10	7.5	07/13	6.5
11033	B73	07/07	7	07/10	7	07/13	6
11030	B73	07/07	7	07/10	7	07/13	6
11036	CML103/B73 NIL-1008	07/20	6	07/24	6	07/27	5
11037	CML277/B73 NIL-1122	07/20	6	07/24	5.5	07/27	5
11025	M162W/B73 NIL-1268A	07/17	6	07/20	5.5	07/24	4
11007	B73	07/20	6.5	07/24	4	07/27	4
11022	B73	07/20	5	07/24	4.5	07/27	4
11023	NC358/B73 NIL-1238A	07/20	4	07/24	3.5	07/27	3
11535	Tx303/B73 NIL-1208	07/20	4	07/24	4	07/27	3
11021	Mo18W/B73 NIL-1113	07/20	3.5	07/24	3	07/27	2.5
11029	CML322/B73 NIL-1018A	07/20	3	07/24	3	07/27	2

#### Field inoculation

Maize plants were inoculated after about 5 weeks of growth at approximately the 8 leaf stage. To initiate the SLB inoculation in the field, maize plants were inoculated with sorghum grains infested with the fungus *C. heterostrophus*. Warm and humid conditions facilitated the subsequent spread of a uniform secondary inoculum, caused by the initial inoculation, throughout the field. The symptoms of SLB infection typically become visible on corn leaves within 4 to 7 days after inoculation. Sermons and Balint-Kurti (2018) give a more detailed description of the inoculation and scoring processes [2].

#### Field trial visual scoring

**Fig 2:**
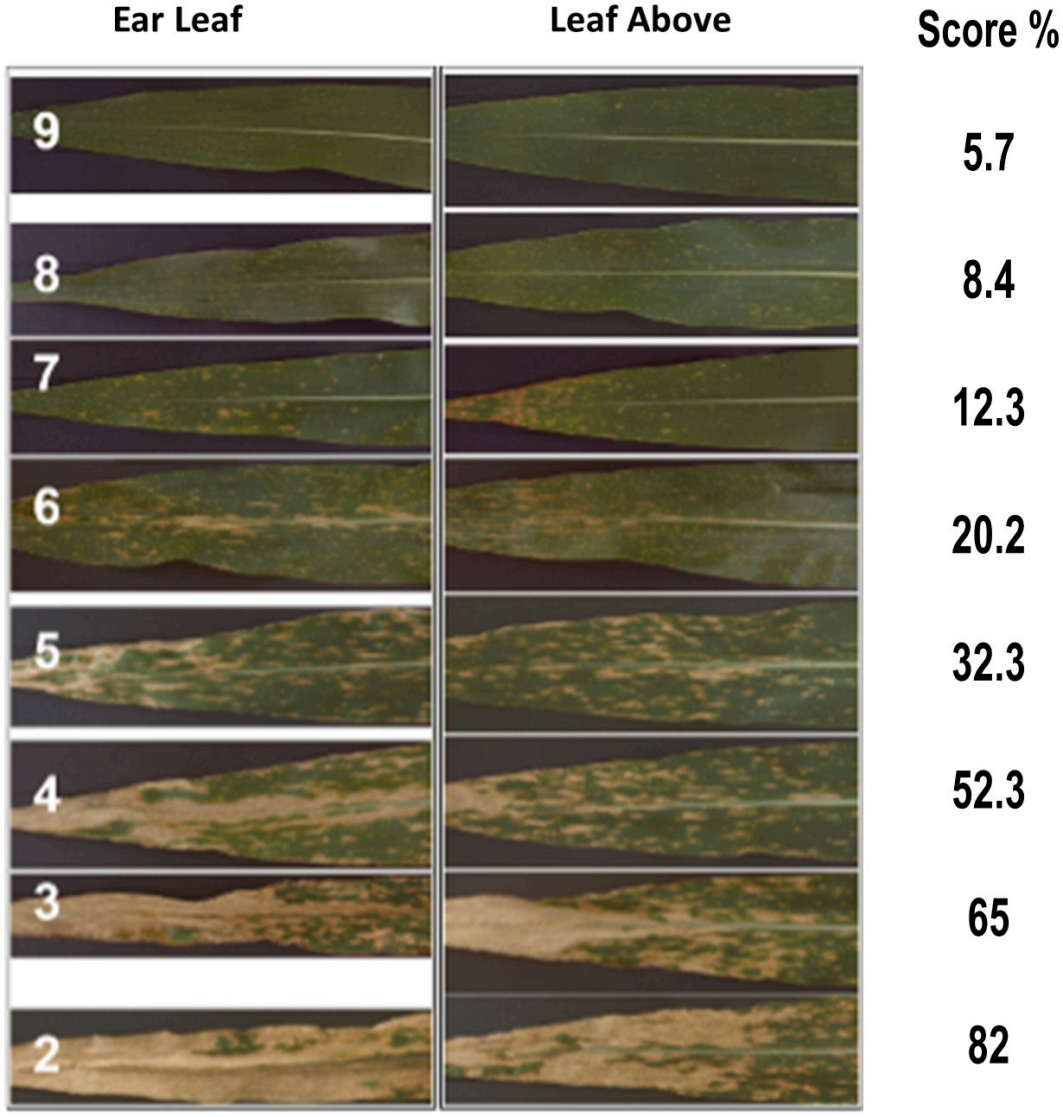
SLB Visual scoring rubric. (Adapted from Supplementary Fig 1 Kump et al. (2011) [26]).

To generate the disease dataset, we collected leaf samples with different stages of SLB infection. These different stages are represented by the visual scores. Flowering occurred approximately 11 weeks after planting and plant disease severity was scored on a 1-9 scale, at intervals of 0.5. Visual scores were based primarily on the appearance of the top-most ear leaf, with 1 being a fully dead leaf and 9 being highly resistant (asymptomatic) [2]. Plants with a score of 8 have few lesion marks on the lower leaves, whereas a score of 7 indicates the appearance of lesions on the ear leaf. If the number of disease indicators increased on the ear leaf, but they did not merge together, a score of 6 was given. A score of 5 was given when lesion spots merged together and formed necrotic areas at the borders. When these necrotic areas reached the leaf above the ear leaf, a score of 4 was given. If the tissues of the leaf above the ear leaf were dead, a visual score of 3 was assigned. Finally, a score of 2 was given to plants that exhibit nearly all dead tissues (Fig 2, adapted from [26]).

#### Field leaf collections

Since the fluorescence spectrometer could not be brought to the field, a maximum of 10 leaf samples per day were brought back to the lab to minimize leaf stress. Leaves were removed from the plants by cutting them at their base, approximately 25 mm from the stalk. To minimize stress during transport, leaves were immediately placed in separate water-containing bags prior to being placed in a cooler (10°*C*). SLB is a highly heritable disease [26], therefore, the representative leaf sample was collected from the field by removing the top-most ear leaves from one of the (typically) 10 plants within a given plot. Control leaves were first collected approximately two months after planting and before field inoculation, in which a total of 75 healthy samples were collected from the two blocks in the field. After inoculation and during the trial period, disease data were collected from lines representing each integer visual score in addition to the intermediate scores of 2.5, 4.5, 6.5, and 7.5. Each line was represented by one leaf. Specifically, a collection of one leaf of scores 4.5, 2.5, and 2, two leaves of scores 8, 7, 6.5, 5, and 3, and three leaves of score 4, and four leaves of scores 6 and 7.5 were collected. This yielded a total of 24 treated corn leaf samples to serve as our *diseased plants* dataset.

### Fluorescence spectroscopy

#### DUV Raman/PL 200

**Fig 3:**
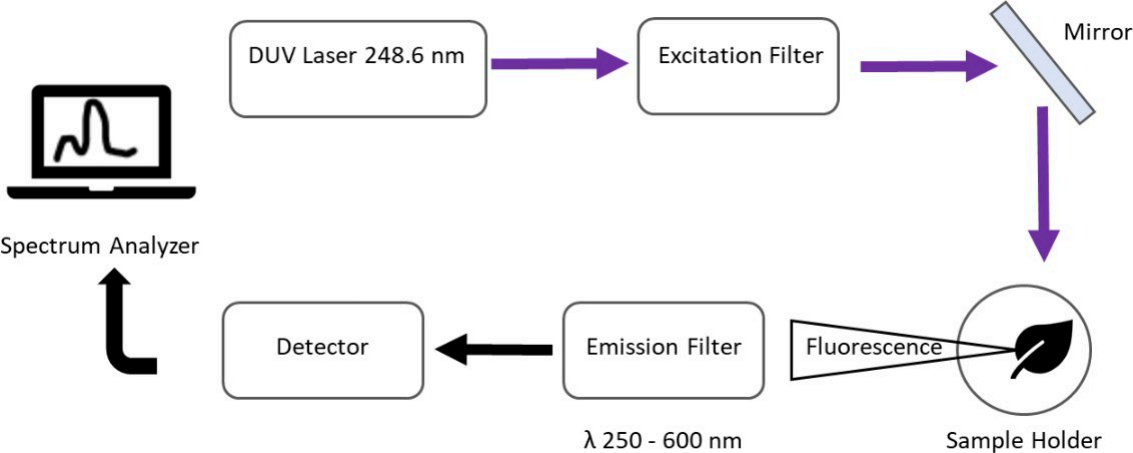
Basic schematic of fluorescence spectroscopy.

A commercial deep UV Raman and fluorescence spectrometer by Photon Systems Inc. (DUV Raman/PL 200) was used to measure the corn leaves’ fluorescence emission spectra. The main motivation for using this instrument was its capability to operate in the DUV region using 248.6 nm excitation wavelength. The laser had a pulse width of ≈35 *μs*, energy of 5 *μj*, and a diameter between 100-200 microns. Fig 3 depicts an illustration of the device’s functionality. First, the DUV laser source transmits through a bandpass filter and is then guided to the sample using a mirror. The fluorescent light emitted from the sample, which has a longer wavelength than the excitation source, is then filtered further to block unwanted signals of the excitation source. Finally, the filtered spectra were measured by the camera’s detector. This system was integrated with spectrum analyzer software to adjust measurement parameters, such as the number of laser pulses, frequency, and the sample holder position.

#### Fluorescence emission spectra collection

Using the fluorescence spectrometer, we have measured the fluorescence emission spectra of healthy and diseased corn leaves in addition to the emission spectrum of C. heterostrophus. To measure the fungus’ spectrum, the PDA plate shown in Fig 1, was unwrapped and placed in the sample holder. Due to the variability of fungal growth on the media, a random sampling consisting of a total of 15 samples was collected. In our experiment, we chose 5 laser pulses with a frequency of 40 Hz to execute the measurements. Similarly, after healthy and diseased corn leaves were in the lab, they were measured within 24 hours by randomly sampling fluorescence emission spectra across each leaf’s surface. Due to the high spatial variability of the lesions, sampling consisted of a total of 50 samples per leaf. Here, we chose 10 laser pulses with a frequency of 40 Hz to execute the measurements.

### Paraquat treatment

The visual scores reflect the symptom levels on maize leaves, specifically the amount of leaf necrosis caused by the disease. To further isolate the causality between the optical emission at 325 nm to the phenotype associated with SLB infection, experiments were performed in which necrosis occurred rapidly due to abiotic chemical interaction. Paraquat, the active toxic chemical in *Gramoxone* herbicide, was used to kill corn leaves rapidly (within a few hours) after application. Liquid Paraquat (100 μMol) was brushed onto a corn leaf. Samples were then collected on day 0 and day 3 after exposure. Then, corn leaf signatures of healthy, day 0, and day 3 after exposure were measured using the DUV fluorescence spectrometer.

### Autoencoders anomaly detection

An Autoencoder is an unsupervised neural network architecture that consists of an encoder and a decoder. The encoder reduces the input dimension by ignoring the noise and the redundant representations of the data. The decoder utilizes this compressed version of the input to reconstruct a representation similar to the original input. The basic architecture of an Autoencoder network consists of the input data *X*, a lower dimension space *Z* known as the hidden layers, and *X’* as an output. In the scope of anomaly detection, the Autoencoder network is a very useful method since it is able to detect abnormalities in the data. More specifically, during training, the network is trained on *good* data and it learns how to reconstruct the input by minimizing the reconstruction error between the input and predicted data, *X* and *X’* respectively. However, when *anomalous* data is passed as an input to the trained model, the network struggles to replicate the original data and raises a reconstruction error with anomalous features [27]. In this study, we called this reconstruction error an AE error.

#### Data preprocessing

Before training the model, it is crucial for the training and testing dataset to be on the same scale. Therefore, the intensity of the treated and healthy spectra was normalized to the maximum peak at 450 nm. Finally, these processed samples were separated into training and testing matrices **X**_**t**_**r** and **X**_**t**_**s** respectively. **X**_**t**_**r** is a matrix comprising 75 healthy corn leaves, whereas **X**_**t**_**s** represents the 24 SLB-infected leaves.

#### Model training and testing

As previously mentioned, the encoder should be trained on the *good* data for the network to efficiently learn the features of the healthy corn spectrum. As shown in 1 [[27]], the activation function is applied to the input vector **X**_**i**_, **X**_**t**_**r**_**i**_ in our case, to get a compressed version, then stored in the hidden layers. Subsequently, the decoder attempts to generate a similar representation of the input using the compressed version in the hidden layer *Z* as seen in 2 [[27]]. Where **X** is the input vector, *Z* is the hidden layer, *n* represents the 75 spectra of the input, and the weight *W* and bias *b* represent the model’s parameters *θ*.

$$\begin{array}{c} Z_{i}=f_{\theta }\left(X\right)=s\left(\Sigma_{j}^{n}{=}_{1}W_{i}jX_{j}+b_{i}\right) \end{array}$$ (1)

$$\begin{array}{c} {\hat{X}}_{i}=g_{\theta }\left(Z\right)=s\left(\Sigma_{j}^{n}{=}_{1}W_{i}jZ_{j}+b_{i}\right) \end{array}$$ (2)

The healthy dataset **X**_**t**_**r** matrix was passed to the MATLAB built-in function *trainAutoencoder*. We determined that using 25 hidden layers and 100 epochs are the optimal hyper-parameters for training. For prediction, the MATLAB built-in function *predict* was used to get the reconstructed matrix **XReconstructed** by inputting **X**_**t**_**s** along with the trained model.

#### Model evaluation

To evaluate our model, we used the absolute error metric to obtain the AE error(*ϵ*) between the predicted data *xReconstructed* and the input data *XTest* per Eq 3. The algorithm identifies an anomaly whenever the error’s magnitude exceeds a user-defined minimum value. Generally, the AE error increases as the deviation between the predicted data *xReconstructed* and the input data *XTest* increases.

$$\begin{array}{c} \epsilon =|XTest-xReconstructed| \end{array}$$ (3)

## Results

### Field visual scores

**Fig 4:**
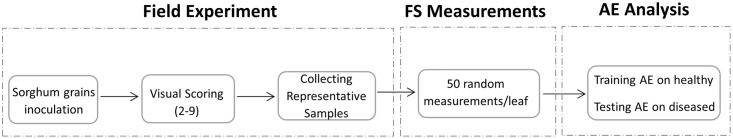
Methods schematic used to quantify the symptoms of SLB-infected leaves.

Visual scores from the field experiments are summarized in Tables 1 and 2. Note that, while a larger population of inbred lines were planted, as discussed in our methods, only 24 leaves, one leaf per line, were selected. Leaf samples were measured within 24 hours after *Date 3*. This date represents the destructive sampling’s date. As previously mentioned, we collected one leaf of scores 4.5, 2.5, and 2, two leaves of scores 8, 7, 6.5, 5, and 3, three leaves of score 4, and four leaves of scores 6 and 7.5. For our analysis, leaves with intermediate scores were binned up to get one leaf with a visual score of 2, three leaves with scores 5, 4, and 3, four leaves of scores 7 and 6, and six leaves of score 8 (Fig 4).

### Fluorescence emission spectra

#### *C. heterostrophus* cultures

**Fig 5:**
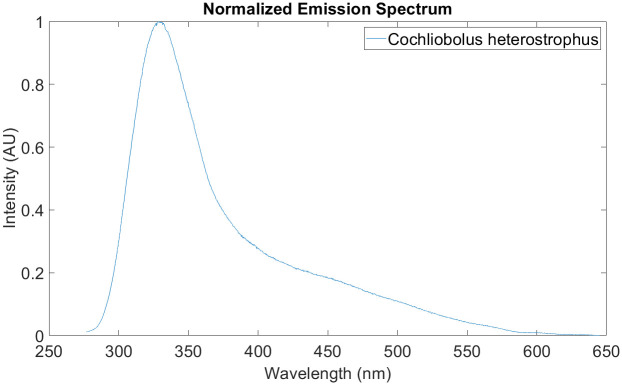
Corn leaf 3 days after Paraquat application.

Measured with DUV RAMAN/PL 200, we took 5 measurements of *C. heterostrphus* grown on a PDA plate to obtain the mean fluorescence signature shown in Fig 5. As shown, *C. heterostrophus* signature has a peak at 325 nm.

#### Paraquat treated tissues

**Fig 6:**
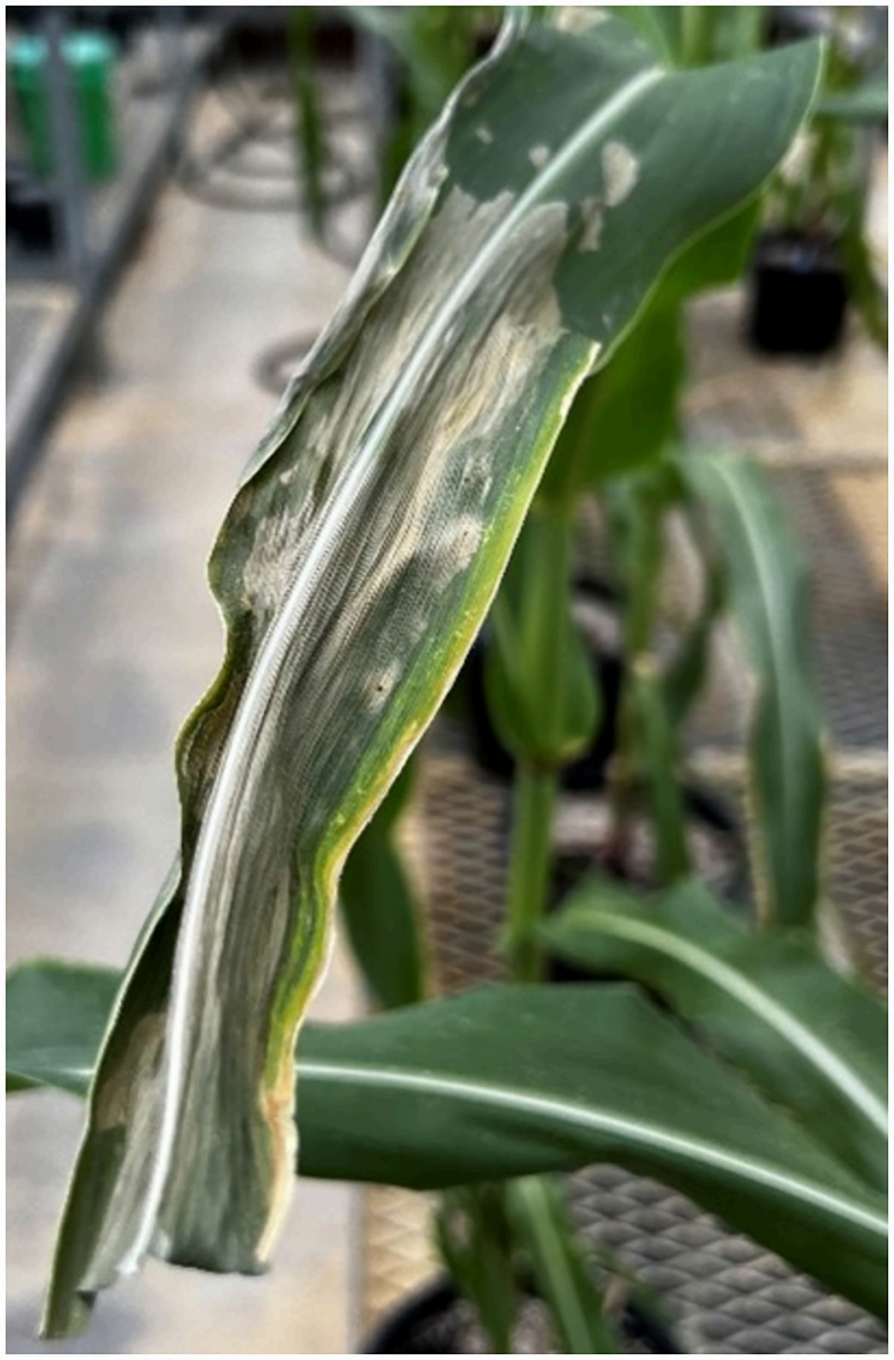
Fluorescence measurement after PQ exposure. (a) Effects of paraquat on corn emission spectra, (b) 325/450 nm fluorescence ratio for (1) healthy, (2) day 0, and (3) day 3.

**Fig 7:**
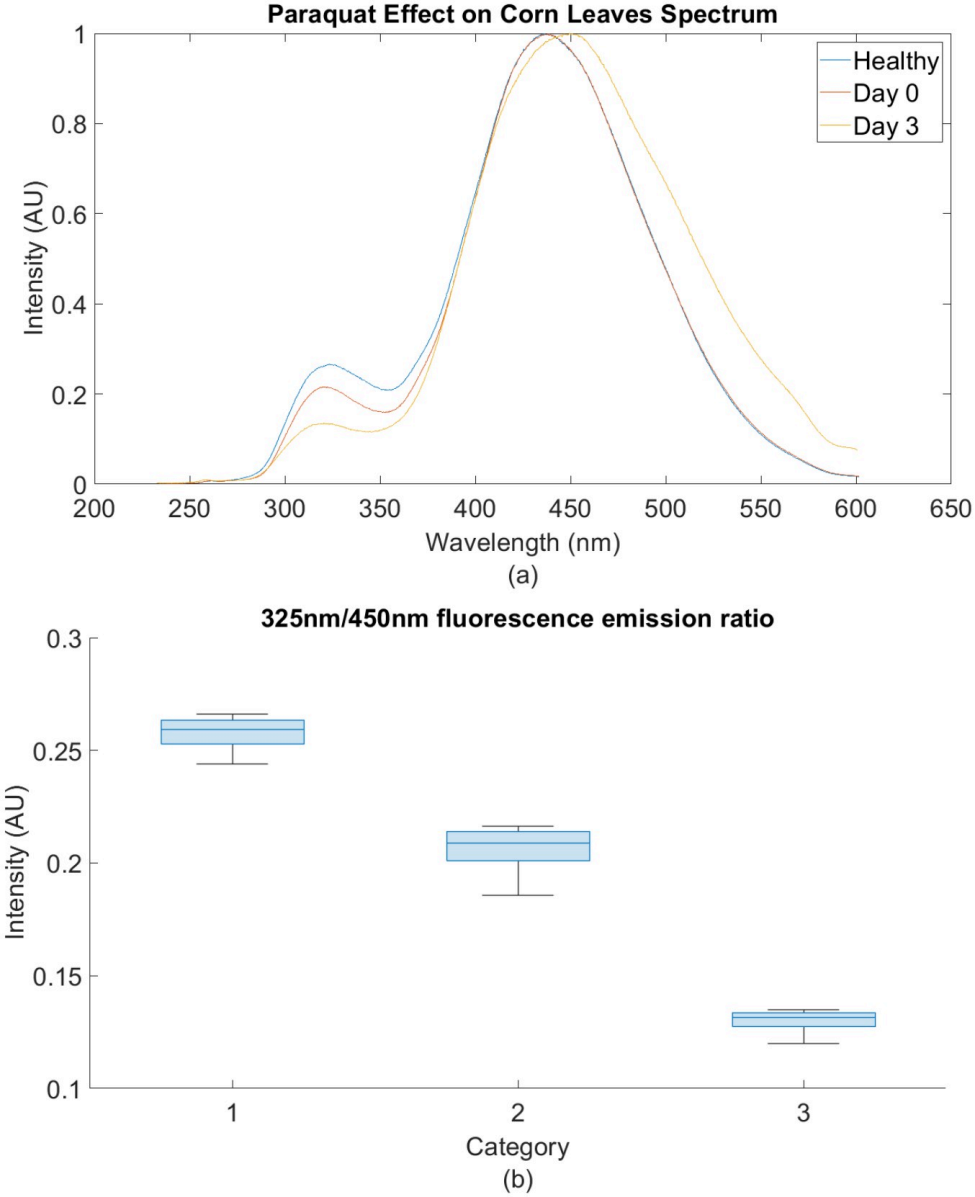
Mean and standard deviation of 50 random fluorescence measurements. (a) Healthy corn leaf, (b) Score 2 corn leaf, and (c) Score 8 corn leaf.

As mentioned earlier, to isolate the causality of the optical emission at 325 nm to the phenotype associated with SLB infection, corn leaves were brushed with Paraquat and then, 50 random measurements of the leaf were taken using DUV RAMAN/PL 200. Fig 6 shows a corn leaf on the third day after Paraquat exposure. Fig 7(a) illustrates the effects of Paraquat on the emission spectrum of corn leaf on day 0 and 3 days after application. Whereas Fig 7(b) shows the change in the intensity of the fluorescence ratio 325/450 nm on day 0 and 3 days after Paraquat exposure.

#### Healthy and diseased tissue

**Fig 8:**
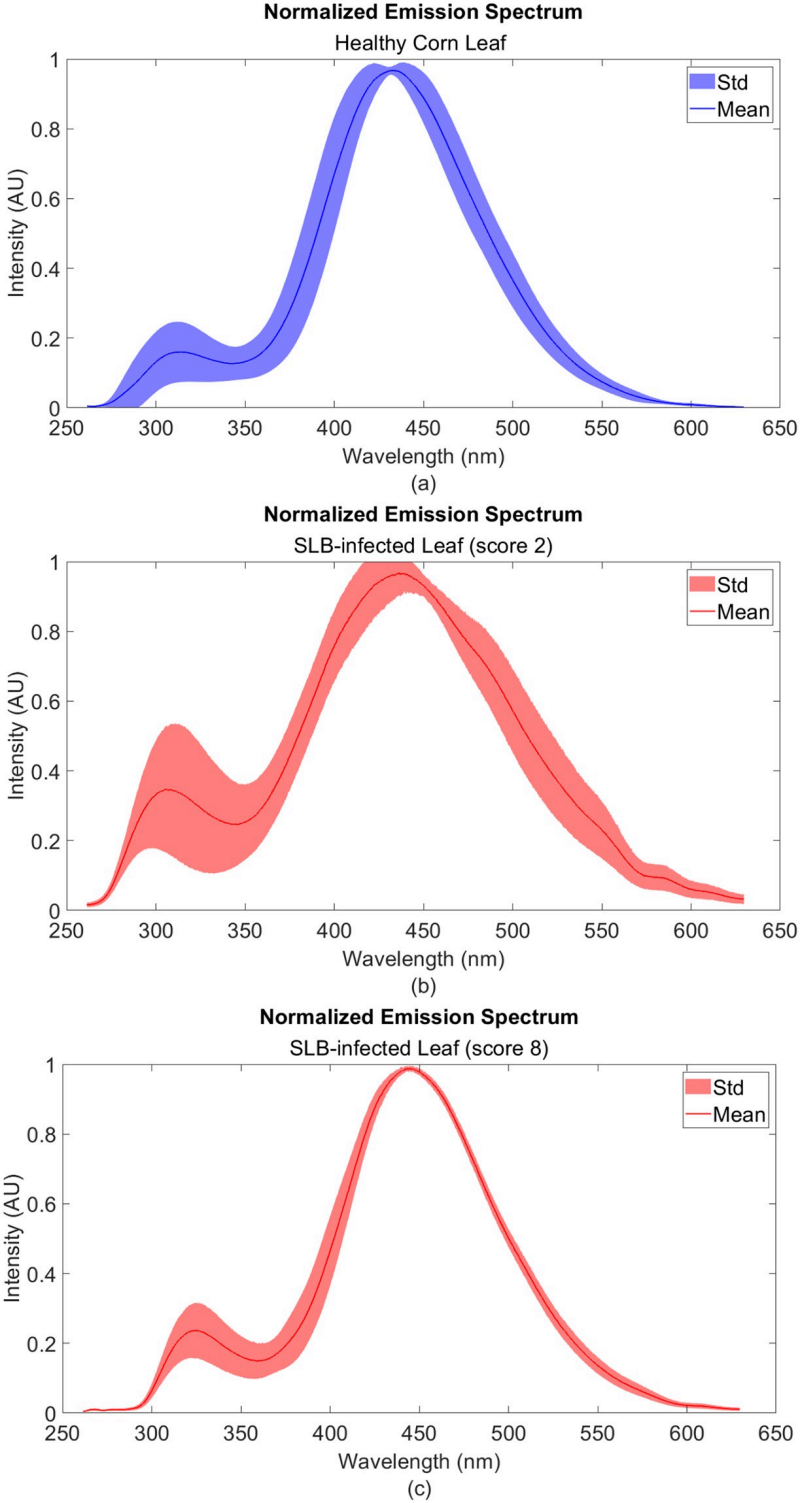
Mean and standard deviation at 325 nm. (a) healthy samples and (b) disease samples.

**Fig 9:**
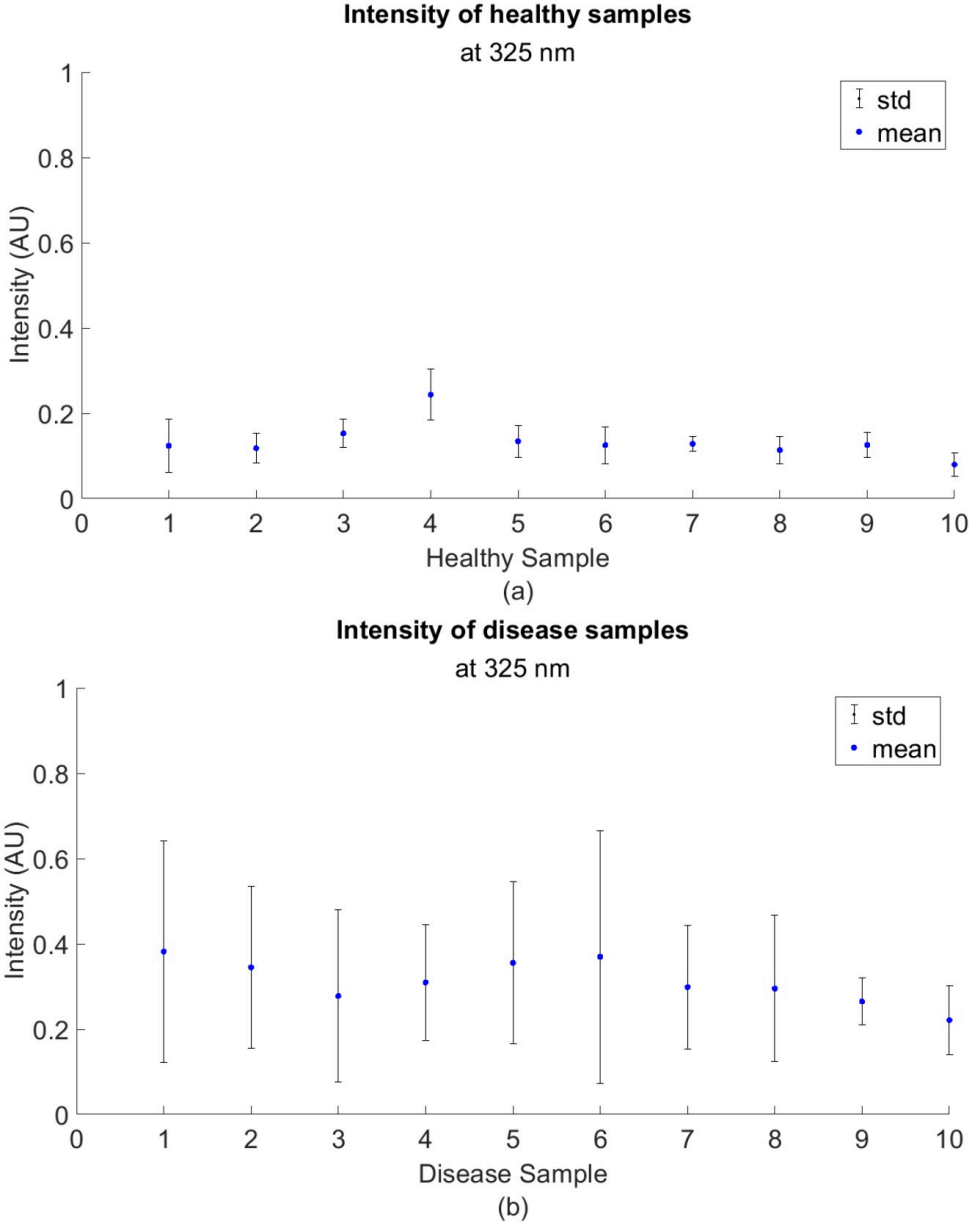
Mean and standard deviation of the 50 measurements for each visual score at 325 nm.

**Fig 10:**
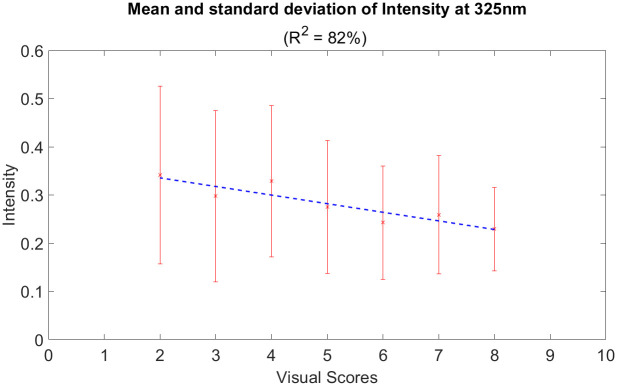
Autoencoder’s model predictions. (a) Score 2 predicted data (b) Score 2 test data (c) Score 8 predicted data (d) Score 8 test data.

Fig 8(a) shows the average fluorescent signature of a healthy corn leaf with prominent peaks at around 325 nm and 450 nm. On the other hand, Fig 8(b) and 8(c) show the fluorescence spectra of SLB-infected corn leaves given a visual score of 2 and 8 respectively. For all graphs, the mean is generated by taking an average of approximately 50 random measurements across the leaf sample. Visual scores like 2 show higher intensity values between the 260 to 350 nm range; whereas, higher scores like 8 possess lower intensity values in the same range. Fig 9 illustrates the statistics of the fluorescence intensity for 10 control and disease samples around 325 nm. In the same range, Fig 10 shows a summary of the statistics of the intensities for each visual score. A linear inverse relationship was obtained with *R*^2^ = 0.82 between the intensity at 325 nm and the visual scores.

### Autoencoder performance on healthy and disease dataset

**Fig 11:**
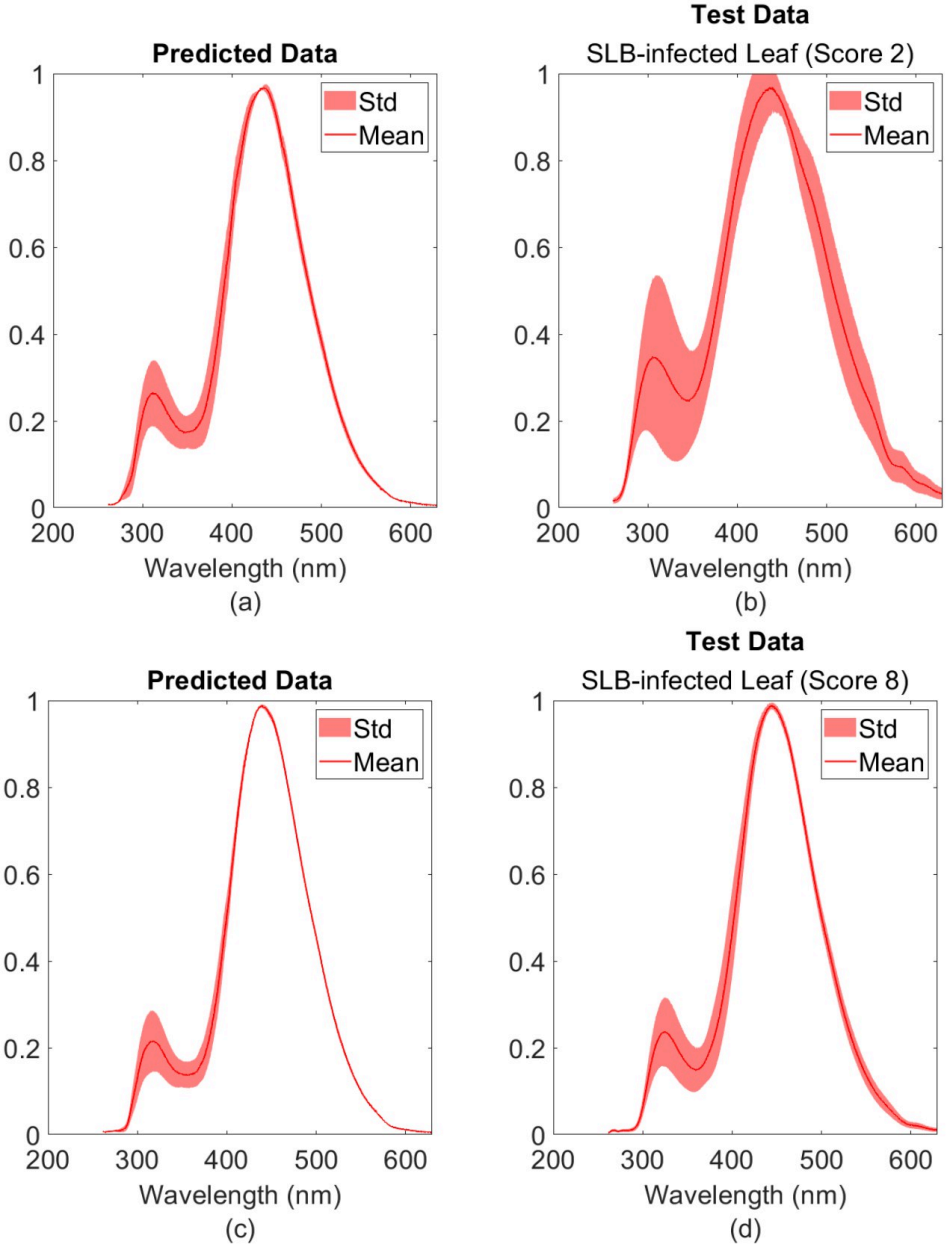
Linear inverse relationship between AE error and VS.

**Fig 12:**
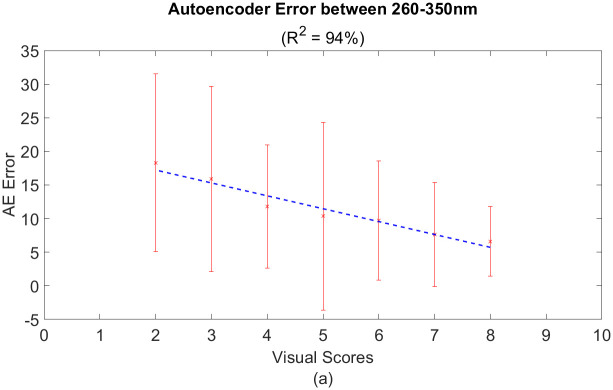


To test the model’s performance, Fig 11 shows the reconstructed, *predicted*, spectra of SLB-infected leaves when visual scores of 8 and 2 are used as inputs into the trained model. This yielded a linear inverse relationship with *R*^2^ = 0.94 between the AE error and the visual scores, as illustrated in Fig 12. In other words, the AE error decreases with higher visual scores and increases when visual scores decrease.

## Discussion

As shown in Fig 5, the fluorescence spectrum of *C. heterostrophus* peaks at around 325 nm. Therefore, we are interested in analyzing this range of wavelengths to study SLB infection in corn plants. Corn plants were treated with Paraquat to isolate the causality between the optical emission at 325 nm to the phenotype associated with SLB infection. Fig 7(a) shows that the fluorescence intensity at 325 nm decreased to 0.2 on day 0 relative to the healthy corn spectra. On the third day after paraquat exposure, the intensity dropped further to reach 0.1. In Fig 7(b), the intensity statistics of the fluorescence ratio at 325/450 nm are shown for 20 measurements of healthy corn leaf, day 0, and day 3 after exposure. Among the three groups, the measurements on day 3 show the lowest intensity at 325 nm with respect to the peak at 450 nm. Since the necrotic areas are apparent on corn leaf sample 6 and the emission of Paraquat-treated samples is decreasing at 325 nm, we can conclude that the contribution of dead tissues at the 325 nm peak is not noticeable.

The intrinsic corn leaf spectrum shown in Fig 7(a) contains two prominent peaks around 325 and 450 nm. In wheat grains, tryptophan contributes to the 320 nm peak when excited with 280 nm wavelength [20]. Although corn is known to be low in tryptophan [27], the possibility of tryptophan contribution at around 320 nm can not be eliminated when excited with a DUV source. The fluorescence peak at 450 nm was consistent with Smeesters et al. (2023) findings of the emission spectrum of maize powder [28]. Cinnamic acids are mainly responsible for the blue-green fluorescence emission from 440 to 530 nm [26].

Fig 8(b) and 8(c) show two fluorescence spectra of SLB-infected leaves given a visual score of 2 and 8 respectively. Comparing that to the healthy leaf spectrum, we can clearly notice the variations in the signatures of these scores. More specifically, there are two prominent variations, the first is at the 280 to 350 nm range, and the second is at the tail between the 550 to 600nm range of the spectrum. We can correlate the increase in intensity at 325 nm to the phenotype of SLB infection since C.heterostrophus has a fluorescence emission maximum also at 325 nm Fig 5. On the other hand, higher scores gave a signature that is less deviant from the healthy leaf spectrum shown in Fig 8(c).

Fig 9 represents the statistics of the intensity for 10 healthy and diseased samples at 325 nm. Clearly, for healthy samples, the mean and standard deviation of the intensities are smaller compared to the diseased samples. In the diseased samples, the phenotype of SLB infection explains the high variability in the mean and standard deviation of the intensity at 325 nm. Moreover, in the same range, Fig 10 shows an inverse linear relationship with *R*^2^ = 0.82 between the intensity values and the visual scores. Since lower visual scores imply a higher infection rate, the mean and standard deviation of the intensity at 325 nm are increasing as the visual scores decrease.

Fig 11 shows the predicted spectrum when a score of 8 and 2 is passed as input to the trained model. From Fig 11(a), it is clear that the model fails to reconstruct anomalous features, especially within the 325 nm range, where *C. heterostrophus* peaks. Whereas, in Fig 11(b), the predicted spectrum is almost identical to the input spectrum since the visual score of 8 has few anomalous features. Therefore, we are interested in calculating the sum of the AE error for wavelengths spanning from 260 to 350 nm. This range was chosen since it captures most of the range associated with the fungus’ peak as shown in Fig 5.

Fig 12 shows the results obtained from calculating the AE error around the 260 to 350 nm range. The linear relationship shows that the AE error and the ground truth labels are inversely proportional. As already stated, our analysis consisted of one leaf with a visual score of 2, three leaves with scores of 5, 4, and 3, four leaves with scores of 7 and 6, and six leaves with a score of 8. For score 2, the point represents the mean and the standard deviation of the AE error taken from the 50 measurements on the leaf. For scores with multiple leaves, the points were generated by taking the mean and the standard deviation of the AE error taken from the 50 measurements per leaf. Then, the results for each leaf were averaged to get a representative mean and standard deviation for each score.

Generally, the mean and standard deviation of the AE error increase as the visual scores decrease. Diseased samples have more necrotic spots compared to healthy samples, causing the standard deviation to vary significantly between the 50 measurements per leaf. On the other hand, healthy samples, or higher visual scores, show relatively smaller mean and standard deviation. The obtained R-squared value is 94% between the Visual Scores and the AE error. This means that the variation of the AE error, the *dependent* variable, can represent approximately 94% of the variations in the visual scores, the *independent* variable.

Few studies utilized DUV FS to determine plant diseases, such as Aflatoxins [29] and Gram-negative bacterial strains [30]. Another study used DUV FS with 280 nm excitation wavelength to detect different mycotoxins in wheat grains, but the toxin emission spectra interfered with the intrinsic emission spectra of grains [22]. In this study, we propose the use of a non-destructive method to quantify the symptoms of fungal infection in SLB-infected corn plants using DUV fluorescence spectroscopy and autoencoder anomaly detection techniques. Although our technique has a limitation in that we are associating the amount of fungus, present on each leaf, to a phenotypic visual score, we were still able to quantify the phenotype associated with SLB infection in corn plants.

This is significant because, per our results shown in Fig 7, the 325 nm emission of dead tissue, caused by the abiotic paraquat treatment, decreased in emission intensity versus time. Conversely, as illustrated in Figs 8 and 10, the optical emission of SLB-infected leaves at 325 nm increased as the infection severity, determined by the visual phenotypic scores, increased. Therefore, we can correlate the increase in intensity, shown in Figs 7 and 9, to the phenotype of SLB infection since C.heterostrophus has a fluorescence emission maximum also at 325 nm (Fig 5). Performing Welch’s t-test with (*P* < 0.05) on the AE error between the 260-350 nm emission, yielded a significant difference between SLB-treated and paraquat-treated samples (Table 3). This result implies that the causality of the fluorescence change at 325 nm is highly likely to be the presence of the fungus, as opposed to a particular phenotype associated with necrotic tissue. Table 3 also shows the statistical difference between two neighboring visual scores.

**Table 3:** Welch’s t-test based on the AE error between 260-350 nm.

Welch’s t-test based on AE error between 260-350 nm.				
Treatment	Mean	SD	Neighboring Significance (*P* < 0.05)	Paraquat Significance (*P* < 0.05)
Score 2	22.12	17.567	ns	s *
Score 3	16.95	17.115	ns	s *
Score 4	17.02	17.366	ns	s *
Score 5	14.77	15.217	s *	s *
Score 6	10.36	11.256	ns	s *
Score 7	9.837	9.6917	s *	s *
Score 8	6.096	5.7885	-	s *
Paraquat	-17.27	3.8891	-	-

Treatment-SLB and abiotic paraquat treated samples; Mean-Average AE error value in the 260-350 nm range; Std-Standard deviation; Neighbor Significance-Significant difference between two neighboring VS; Paraquat Significance-Significant difference between SLB and paraquat treated samples.

Further efforts are needed to correlate the optical emission spectrum of SLB-infected leaves to the presence of C. heterostrophus as based on e.g., fungal spore concentration and spatial localization (as determined by microscopy) within the lesion. Since measurements were taken in the field, it is also possible that the technique is detecting other fungal species, beyond C. heterostrophus, that are colonizing the tissues behind the infection. Further work is needed to resolve this, but overall, we have demonstrated fluorescence emission with a negative slope using a spectral region that should have a positive correlation to the necrotic tissue.

## Conclusion

We demonstrate the use of a commercial DUV fluorescence spectroscopy to quantify the symptoms of fungal infection in SLB-infected corn leaves. The fungus, healthy, and SLB-infected corn leaves emission spectra were measured using an excitation wavelength of 248.6 nm. Healthy and SLB-infected corn leaves showed two peaks one at 450 nm and the other between 320-325 nm; whereas *C. heterostrophus* showed a dominant peak at 325 nm. SLB-infected leaves gave a higher fluorescence response at 325 nm than healthy leaves. We correlate this increase in intensity to the phenotype of SLB infection since C.heterostrophus has a fluorescence emission maximum also at 325 nm. Moreover, the visual scores were used as a ground truth label to rate the severity of SLB infection in the disease samples. We achieved a linear inverse relationship between the intensity response at 325 nm and the visual scores (R2 = 0.82 and RMS error of 0.0166).

As a more robust method of quantification, we demonstrate the use of an anomaly detection model based on an autoencoder error. The AE error was used as a metric to quantify the symptoms of fungal infection in SLB-infected leaves between the 260-350 nm range. This gave a linear inverse relationship between the AE error and the visual scores (R2 = 0.94 and RMS error of 0.935). One challenge we experienced involved having to take samples back to the lab for quantification. Since this was a limitation of our commercial sensor, we envision having a more portable device, that leverages deep UV LEDs, to allow for trace foliar fungal detection high-throughput imaging. Ultimately, autoencoders, combined with UV fluorescence spectroscopy, can enable a sensitive quantification method for detecting SLB infection in corn plants.
